# Investigation of Phenolic Compounds and Antioxidant Activity of *Sorbaria pallasii* (Rosaceae) Microshoots Grown In Vitro

**DOI:** 10.3390/life13020557

**Published:** 2023-02-16

**Authors:** Titiana V. Zheleznichenko, Tatiana N. Veklich, Vera A. Kostikova

**Affiliations:** 1 Central Siberian Botanical Garden, Siberian Branch of Russian Academy of Sciences (CSBG SB RAS), 630090 Novosibirsk, Russia; 2 Amur Branch of Botanical Garden-Institute, Far Eastern Branch of Russian Academy of Sciences, 675000 Blagoveshchensk, Russia

**Keywords:** *Sorbaria pallasii*, HPLC, catechin, phenolcarboxylic acid, tannin, flavonoid

## Abstract

*Sorbaria pallasii* is an endemic species of the Far East and Siberia and grows along the Goltsy altitudinal belt. Data on micropropagation and phytochemical characteristics of this plant are not available, probably because of the inaccessibility of the plant material. Morphogenesis initiation from flower buds of *S. pallasii* in vitro and micropropagation were performed here in the Murashige and Skoog medium supplemented with 5.0 µM 6-benzylaminopurine and 0.0–1.0 µM α-naphthylacetic acid; elongation was implemented in the same medium without the hormones. A well-growing sterile culture of *S. pallasii* was obtained; the number of microshoots per explant reached 5.7 ± 1.2. Phytochemical analyses of in vitro propagated *S. pallasii* detected 2,2-diphenyl-1-picrylhydrazyl (DPPH) radical scavenging activity in a water-ethanol extract from its microshoots and revealed phenolic compounds in it. The phenolic compounds that likely contribute to its biological activity are tannins (74.9 mg/g), phenolcarboxylic acids (30.8 mg/g), and catechins (13.3 mg/g). In the microshoot extract, high-performance liquid chromatography identified three catechins. Microshoots showed the highest concentration of (±)-catechin (3.03 mg/(g of absolutely dry mass; ADM)). Concentrations of epigallocatechin gallate (0.38 mg/(g of ADM)) and (−)-epicatechin (0.55 mg/(g of ADM)) were significantly lower. This study paves the way for further biotechnological and phytochemical research on *S. pallasii*.

## 1. Introduction

Biologically active substances of plants, including secondary metabolites, are a valuable resource for drug development and formulations [1]. Research into active ingredients of plants is underway for both direct use of secondary metabolites as drugs and for their use as precursors for subsequent chemical modifications [2]. The demand for biologically active substances of plant origin grows globally every year [3]; this is especially true for the search for effective therapeutics against COVID-19 [4,5].

**Figure 1:**
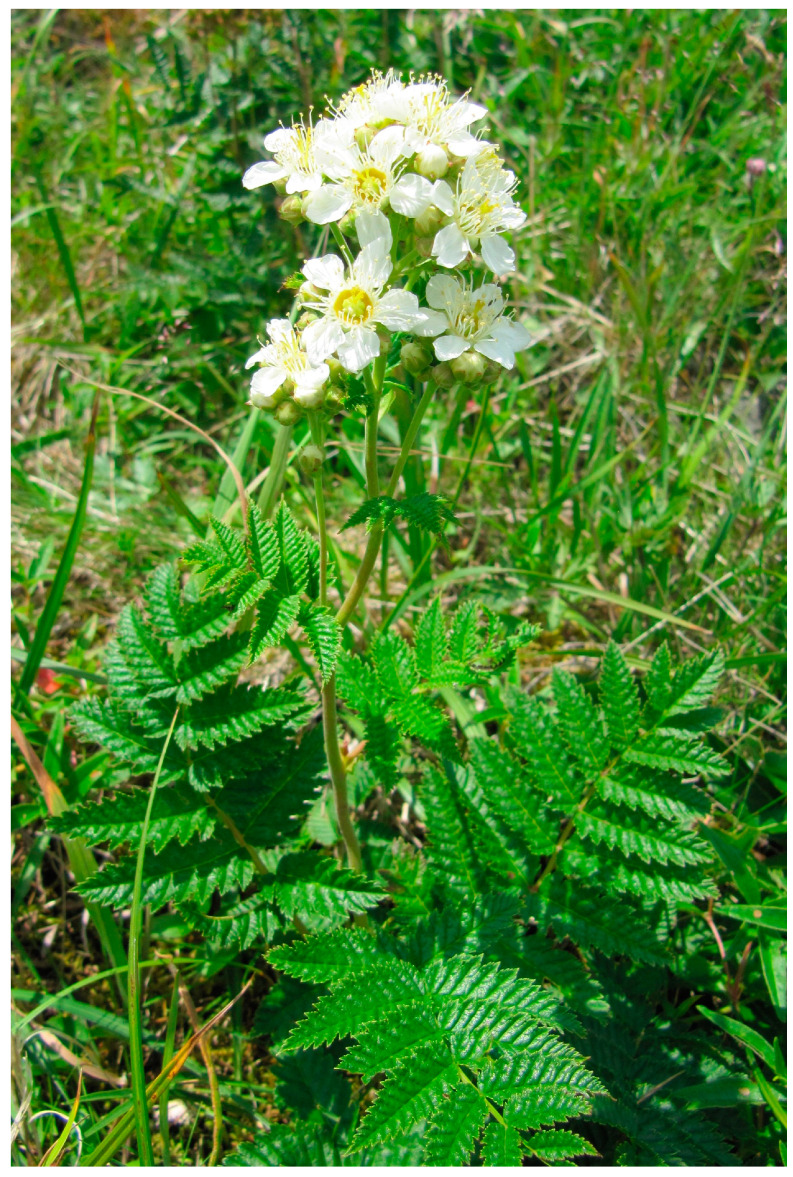
*S. pallasii* (photo by Tatiana N. Veklich).

Conventional medicine currently takes advantage of only ~5% of all the species of higher plants that have been studied [6]. Nonetheless, poorly studied or unexplored plant species are a great potential source of new biologically active compounds [7]. One of these species is *Sorbaria pallasii* (G.Don) Pojark. [= *S. grandiflora* (Sweet) Maxim.]. It is a semi-shrub up to 50 cm high that belongs to the family Rosaceae Juss (Figure 1). Young shoots are brownish, glabrous, or finely pubescent with yellowish branched hairs, and older shoots have peeling bark. Leaves are imparipinnate, up to 15 cm long, of 9–15 pairs of leaflets, dark green, glabrous, or often pubescent. Flowers are in small obovate panicles and white, pinkish, or creamy white, up to 1.5 cm in diameter. Fruits are pubescent follicles with small seeds. The follicular fruit contains small seeds. The species grows along the alpine mountain belt, on bald mountains and stony slopes and placers, sometimes among thickets of dwarf pine. *S. pallasii* is endemic to Eastern Siberia and the Far East [8,9,10] and is included in the endangered species list (Red Book) of Magadan Oblast [11].

The literature data on concentrations of biologically active substances in *S. pallasii* are fragmentary. High-performance liquid chromatography (HPLC) has identified at least 22 phenolic compounds (PCs) in water-ethanol extracts from the leaves of *S. pallasii* and not less than 28 PCs in water-ethanol extracts from its inflorescences [12]. These include two acids (chlorogenic and *p*-hydroxybenzoic) and five flavonols (hyperoside, isoquercitrin, quercitrin, kaempferol, and astragalin) [12]. Data on biological activity of *S. pallasii* are not yet available. On the contrary, another representative of the genus *Sorbaria* (Ser.) A. Braun—*S. sorbifolia* (L.) A. Braun—which grows in Russia, Mongolia, Japan, and China, has been studied sufficiently well. It has been found to improve blood flow, eliminate stasis, reduce swelling, and relieve pain; it is used to treat fractures, bruises, and rheumatoid arthritis in traditional Chinese medicine [13]. A decoction of *S. sorbifolia* is employed to treat diarrhea and rheumatoid arthritis in Amur Oblast and Transbaikalia. The plant is used to cure skin tuberculosis and other skin diseases [14]. An ethyl acetate extract from the above-ground part of *S. sorbifolia* possesses immunomodulatory properties and enhances an antitumor effect of chemotherapeutic drugs against inoculated carcinoma 180 [15]. A dry extract from *S. sorbifolia* inflorescences has been prepared that has antiviral and antioxidant activity [16]. Scientists across the globe have revealed antitumor, anti-inflammatory, antimicrobial, antimelanogenic, and antioxidant properties of extracts from *Sorbaria* species [17,18,19,20,21,22]. In their phytochemical studies on *Sorbaria*, they have isolated triterpenoids, such as cucurbitacin [23], flavonoids, phenolcarboxylic acids, cyanoglycosides, and chromone derivatives [18,24,25], and triterpene acids of ursane and oleanane series [26]. Data on *S. pallasii* are scarce probably because it grows in hard to reach locations, e.g., on mountain slopes above the tree line. Another species, *S. sorbifolia,* has been extensively introduced throughout Russia and abroad; it grows in easy to reach places, and its habitat does not extend high into the mountains.

The pharmaceutical industry is highly dependent on medicinal plants, and as a consequence of its excessive activities, many wild plant species are endangered or extinct [27]. An alternative way to obtain secondary metabolites of plant origin is the culturing of plant cells and tissues (in vitro). The in vitro cultivation technique may help not only to preserve wild plants in nature but also to prepare standard plant materials from hard-to-propagate or poorly accessible plants for the pharmaceutical industry.

The aims of this study were to create a well-growing sterile culture of *S. pallasii* microshoots under in vitro conditions and to perform its phytochemical analyses.

## 2. Materials and Methods

### 2.1. Induction of Clonal Micropropagation and Preparation of the Extract

The material for introduction into the in vitro culture was collected in late September 2021. *S. pallasii* plants grew in the experimental plot of the Laboratory of Phytochemistry, the Central Siberian Botanical Garden (CSBG) SB RAS. They had been introduced in 2019 from the Zeya State Nature Reserve (Amur Oblast, Zeya District), in the upper reaches of the Big Erakingra River of the subalpine zone of the Tukuringra Ridge (54°07′05.7″ N, 126°56′02.4″ E, 1094 m above sea level). The plants grew on rocky debris overgrown with moss. The explants were flower buds of a generative plant. The shoots were cut into segments (one bud per segment) and sterilized in a 0.2% HgCl_2_ solution for 15 min and then washed three times with sterile distilled water for 10 min. Next, the outer scales and some rudimentary leaves were aseptically removed from the buds by means of tweezers and a scalpel; the buds were separated from the branches and placed onto the Murashige and Skoog (MS) agar medium [28] supplemented with 5.0 µM 6-benzylaminopurine (BA; Sigma-Aldrich, St. Louis, MO, USA) to initiate morphogenesis. Each bud was cultured separately in a penicillin vial for 30 days to reduce contamination. The resulting primary microshoots were cut into single-node segments, and the leaves and unexpanded buds were also separated and transferred to the micropropagation medium. The medium was supplemented with the following plant growth regulators (PGRs): 5.0 µM BA in combination with 0.0–1.0 µM α-naphthylacetic acid (α-NAA; Sigma-Aldrich, St. Louis, MO, USA); the passage lasted 30 days. For microshoot elongation, the plant material was cultured in the hormone-free medium of the same mineral composition. The passage lasted 30 days. Elongated microshoots were cut into single-node segments and cultivated either in a solid or liquid medium (no agar) of the same mineral and hormonal compositon and in corresponding PGR-free media.

All the media were supplemented with 3% of sucrose (Shostka Chemica l Reagents Plant, Shostka, Ukraine), and pH was adjusted to 5.8 before autoclaving (sterilization). The solid media contained 0.6% of agar (PanReac, Barcelona, Spain). The media were autoclaved at 121 °C for 20 min. PGRs were added aseptically after sterilization and cooling of the media to 40 °C.

The culturing was performed under a 16 h photoperiod at 40 μmol m^−2^ s^−1^ light intensity provided by cool white fluorescent lamps at 23 ± 2 °C. In the liquid media, the culturing was performed in 100 mL Erlenmeyer flasks using a shaker (Elmi, S-3-02 L, Latvia) at 100 rpm; the volume of the medium in the flask was 20 mL.

Phytochemical analyses of the microshoots were performed after their cultivation in the hormone-free MS agar medium for three passages.

### 2.2. Identification and Quantitation of Phenolic Compounds (PCs) in Water-Ethanol Extracts Prepared from the Microshoots

#### 2.2.1. Extract Preparation

To assess the levels of PCs and antioxidant activity of *S. pallasii* microshoots, the plant material was air-dried completely at room temperature in shade and weighed. Then, the dry material was shredded into 2–3 mm pieces and blended, and representative samples were chosen. PCs were identified and quantified in an extract obtained by incubation with 70% ethanol in a water bath with reflux at 70 °C. The extract was prepared at the 1:500 ratio of the raw material to the solvent.

#### 2.2.2. Quantification of PCs

The total level of PCs was determined using the Folin–Ciocalteu reagent [29]. Briefly, 0.5 mL of the extract, 2.5 mL of the Folin–Ciocalteu reagent (diluted 1:10 with distilled water), and 2 mL of a 7.5% aqueous sodium carbonate solution were combined and shaken thoroughly. The mixture was kept in a water bath for 15 min at 45 °C. Absorption was measured at a wavelength of 765 nm using an SF-56 spectrophotometer (Lomo, St. Petersburg, Russia). A blank sample consisting of distilled water and the reagents served as a control. Gallic acid was used as a reference compound.

#### 2.2.3. The Total Flavonoid Content

Flavonoids were quantified by the spectrophotometric aluminum chloride technique [30]. Briefly, 0.5 mL of 2% aluminum chloride (AlCl_3_) in ethanol was mixed with the same volume of the plant extract. After 1 h, absorbance readings at 415 nm against a blank (ethanol) were acquired. Optical density of the mixture was measured on the SF-56 spectrophotometer (Lomo, St. Petersburg, Russia). The concentration of each flavonoid was calculated with the help of a calibration curve plotted for rutin (Sigma-Aldrich).

#### 2.2.4. Quantitation of Total Phenolic Acids

The total level of phenolic acids was determined by means of Arnov’s reagent [31,32]. Briefly, 1 mL of the extract, 5 mL of distilled water, 1 mL of hydrochloric acid (0.1 mol), 1 mL of Arnov’s reagent (10.0 g of sodium molybdate and 10.0 g of sodium nitrate in 100.0 mL of water), and 1 mL of sodium hydroxide (1 mol) were mixed, the volume was adjusted to 10 mL with distilled water, and optical density was measured immediately at 490 nm on the SF-56 spectrophotometer (Lomo, St. Petersburg, Russia). A blank sample consisting of distilled water and the reagents was used as a control, and caffeic acid served as a reference.

#### 2.2.5. Quantification of Tannins

This assay of tannins (hydrolyzable tannins) was performed using the method proposed by L.M. Fedoseeva [33]. Briefly, 10 mL of the extract was transferred into a 100 mL volumetric flask, and 10 mL of a 2% aqueous solution of ammonium molybdate was introduced. The flask’s content was brought to the nominal volume with purified water and incubated for 15 min. The intensity of the resulting color was measured using the SF-56 spectrophotometer (Lomo, St. Petersburg, Russia) at 420 nm in a 1-cm light-path cuvette. A government standard sample of tannin (Sigma, St. Louis, MO, USA) was utilized.

#### 2.2.6. Quantification of Catechins

The concentration of catechins was determined spectrophotometrically by the method based on the ability of catechins to produce a crimson color in a solution of vanillin in concentrated hydrochloric acid [34,35]. Briefly, 0.8 mL of the extract was placed into two test tubes. Next, 4 mL of a 1% solution of vanillin in concentrated hydrochloric acid was poured into one of them, and the volumes were adjusted to 5 mL in both tubes with concentrated hydrochloric acid. The tube without vanillin served as a control. In the presence of catechins, the sample turned pink, raspberry, or orange-red. After 5 min, the intensity of the colors was measured through the use of the SF-56 spectrophotometer (Lomo, St. Petersburg, Russia) at 504 nm in a cuvette with a light path of 1 cm. The standard curve was constructed on the basis of (±)-catechin (Sigma, St. Louis, MO, USA).

#### 2.2.7. Quantitation of Individual PCs via HPLC

This analysis was performed using an Agilent 1200 HPLC system, which included a Zorbax SB-C18 column (5 mm, 4.6 × 150 mm) and was equipped with a diode array detector and a ChemStation system for collection and processing of chromatographic data (Agilent Technology, Santa Clara, CA, USA). The separation was conducted under the following conditions: in the mobile phase, the concentration of methanol in the solution of phosphoric acid (0.1%) was changed from 22% to 100% during 36 min. The eluent flow rate was 1 mL/min, column temperature was 26 °C, and sample volume was 10 µL; the detection was conducted at wavelengths 254, 210, 230, 280, 315, 340, and 360 nm. Quantification of individual compounds in the plant extract samples was conducted by the external standard method. For detection of catechins in the plant extract, standard samples of (±)-catechin (Sigma-Aldrich, Taufkirchen, Germany Germany), (−)-epicatechin (Serva, Heidelberg, Germany), and epigallocatechin gallate (Teavigo, Kaiseraugst, Switzerland) were employed, from which standard solutions were prepared (10 μg/mL).

### 2.3. Assessment of an Antiradical Activity

Free radical scavenging capacity of the samples was determined by the 2,2-diphenyl-1-picrylhydrazyl (DPPH) method [36,37] with modifications. Briefly, 2 mL of the extract (diluted with 70% ethanol to concentrations in the range of 50–800 µg/mL) was mixed with 3 mL of a DPPH solution (62 mg/mL in ethanol). After 30 min incubation in the dark at room temperature, optical density (A) was measured at 517 nm as compared to a blank sample. The free radical scavenging activity was calculated as percentage inhibition using the following formula:

I% = (A_blank_ − A_sample_/A_blank_) × 100,

where A_blank_ is optical density of the control solution (containing all reagents except the tested extract), and A_sample_ is optical density of the sample.

The results were expressed in IC_50_ (toward DPPH) defined as the concentration of an antioxidant that causes a 50% loss of DPPH in the DPPH radical scavenging activity assay. Ultimately, 6-Hydroxy-2,5,7,8-tetramethylchroman-2-carboxylic acid (trolox) and ascorbic acid solutions (2.5–50.0 mg/mL) were used as positive controls.

### 2.4. Chemicals

All chemicals were of HPLC or analytical grade.

### 2.5. Statistical Analysis

The data were statistically processed by conventional methods using STATISTICA 6.0 and GraphPad Prism v.6.01 software (GraphPad Software, Boston, MA, USA). All the phytochemical experiments were set up with two biological replicates and three technical replicates per treatment. The data are presented as the mean ± standard deviation. The results were expressed in mg/(g of absolutely dry mass [ADM]).

## 3. Results and Discussion

### 3.1. Induction of Morphogenesis and Development of Microshoots

**Figure 2:**
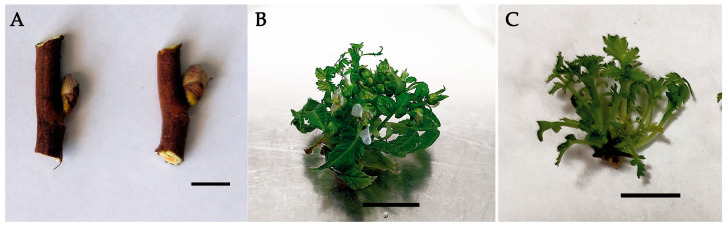
*S. pallasii* photos. (**A**) Buds of an intact plant; (**B**) primary microshoots; (**C**) development of microshoots after 30 days of culturing in the MS medium supplemented with 5.0 µM BA.

Surface sterilization of the plant material performed using HgCl_2_ and subsequent culturing of each explant (Figure 2A) resulted in a high yield (that reached 70%) of aseptic explants. Because *S. pallasii* was introduced into in vitro culture for the first time, the MS medium, the most common culture medium for this purpose, was used as a mineral base. BA and α-NAA (PGRs most commonly used for clonal micropropagation experiments) were utilized for the induction of morphogenesis [38,39,40,41,42]. After 2 weeks of the culturing, initiation of morphogenesis was observed, and a month later, a single 20-mm-high microshoot with flower buds and leaves developed from the explants (generative buds; Figure 2B). The frequency of regeneration of noncontaminated material reached 94%. Primary microshoots were shredded into pieces, including buds, leaves, and single-node stem segments. They were cultured for a month in the micropropagation medium: either MS plus 5.0 µM BA or MS plus 5.0 µM BA in combination with 1.0 µM α-NAA. During culturing of leaves and buds, no further morphogenesis was registered. Regeneration was observed only in single-node segments in culture media of both hormonal compositions (Figure 2C). In the medium with cytokinin (5.0 µM BA), the number of microshoots per explant was 5.7 ± 1.2, and in the medium with the cytokinin and auxin (5.0 µM BA and 1.0 µM α-NAA), it was slightly higher: 5.8 ± 1.4. The height of the microshoots on the medium containing BA was 9.3 ± 3.9 mm, and the number of leaves per microshoot was 4 ± 1. On the medium containing the auxin, the height of the microshoots was greater (10.8 ± 3.3), but the number of leaves was slightly lower (3.9 ± 0.9). Nevertheless, chlorosis was detected in the experiment with the medium containing the auxin and cytokinin. It should be noted that the choice of the PGR concentration, combination, and ratio for the preparation of the medium not only is species-specific but also depends on the genotype of the specimen under study.

For example, a combination of 5.0 µM BA and 2.5 µM α-NAA has been found to be appropriate for the most successful microshooting in cultures of the rare species *Rhodiola rosea* [40]. In addition, a combination of 15.0 µM BA and 5.0 µM α-NAA has turned out to be optimal for clonal micropropagation of *Banana* cv. *Karpuravalli* [39]. At the same time, a similar combination of BA and α-NAA used in in vitro culture of tree peony causes mass formation of somatic embryos [41]. In the present study, we chose the PGR combination and ratio based on our previous experiments on in vitro micropropagation of *Spiraea betulifolia* ssp. *aemiliana* [42]; this PGR ratio was optimal for *Spiraea betulifolia* but quite unsuitable for *S. pallasii*; therefore, *S. pallasii* was further cultured in the medium containing only the cytokinin (BA).

In vitro culturing in a liquid medium is worthwhile because it is more economically advantageous and does not require expensive gelling agents. According to some data, culturing in liquid media increases the productivity and growth rates of plantlets in vitro [38,43]. Judging by other evidence, not all plant species can be cultured successfully under these conditions, which often cause vitrification of the plant material [44]. It is also reported that culturing in a liquid medium can be successful if a plant material floats on the surface of the culture medium [45]. During our micropropagation of *S. pallasii* in the liquid medium, either with or without the PGR(s), the plant material was immersed to the bottom of the culture vessel. This led to microshoot thickening, chlorosis, and local necrosis; therefore, *S. pallasii* was further incubated in the solid (agar-containing) medium.

### 3.2. Phytochemical Characterization of the S. pallasii Microshoots

**Figure 3:**
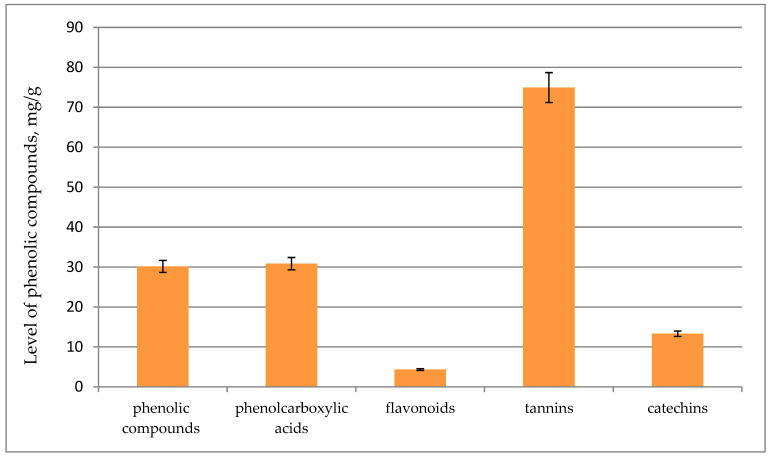
Concentrations of PCs in *S. pallasii* microshoots.

PCs are secondary metabolites synthesized in response to stressful conditions in order to perform some physiological functions in plants. They protect from ultraviolet (UV) radiation and predators and participate in plant growth and plant responses to environmental stressors, including an injury, a pathogen invasion, mineral deficiency, and temperature stress [46,47,48]. PCs are among the main compounds that impart antioxidant properties to plants [49]. Excessive production of free radicals in the human body can cause such illnesses as cancer, atherosclerosis, Alzheimer’s disease, Parkinson’s disease, and cerebrovascular and cardiovascular diseases. A number of studies have revealed a preventive effect of PCs against these diseases [49,50,51]. In vitro systems of cultivation of various plant species can be an alternative source of polyphenolic compounds having a strong antioxidant effect, even stronger than that of intact plants [52,53]. Here, we investigated concentrations of PCs in the water-ethanol extract of *S. pallasii* microshoots (Figure 3). The extract was found to contain phenolcarboxylic acids at 30.8 mg/(g of ADM), whereas the content of flavonols (4.3 mg/[g of ADM]) was modest: seven-fold lower than that of phenolcarboxylic acids. The highest concentration of tannins (74.9 mg/(g of ADM)) and a relatively high level of catechins (13.3 mg/(g of ADM)) were detected in microshoots. The total phenolic content determined by the Folin–Ciocalteu assay was 30.2 mg/(g of ADM).

**Figure 4:**
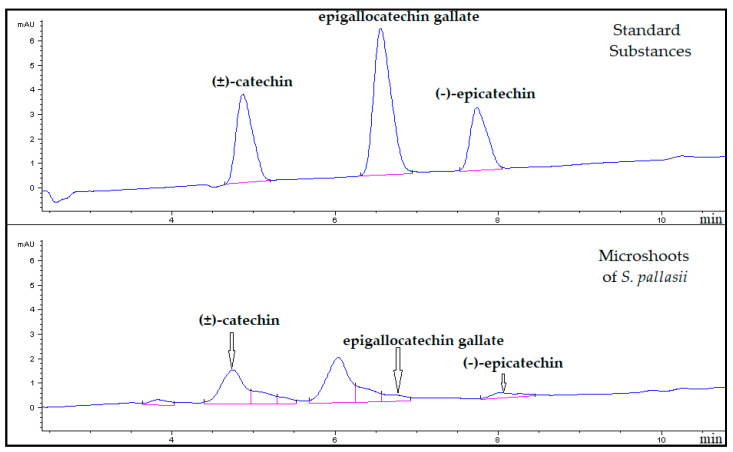
Chromatograms of standard substances and of the 70% ethanol extract from the microshoots of *S. pallasii* at 280 nm. On the *X*-axis: retention time, min; on the *Y*-axis: the detector signal, units of optical density.

Catechins or flavan-3-ols belong to the flavonoid class. The catechin molecule contains two asymmetric carbon atoms on the pyran ring (C2 and C3), and hence each catechin can have four isomers and two racemates. A characteristic feature of catechins is the ability to accept a gallic acid residue with the formation of esters: gallates. *Camelia sinensis* and its in vitro cultures are a rich source of nongallated and gallated catechins, i.e., catechin gallates [46,54,55]. Individual catechins have been identified in representatives of the genus *Sorbaria* previously. For example, (±)-catechin and (−)-epicatechin have been detected in the above-ground part of *S. sorbifolia* var. *Stellipa* Maxim. [56] In our current work, based on the UV spectra and a comparison of retention times of peaks of substances in chromatograms of the analyzed samples with those of the standard samples, three catechins were identified in *S. pallasii* microshoots by HPLC: (±)-catechin (λ_max_ = 280 nm; t_R_ = 4.8 min), epigallocatechin gallate (λ_max_ = 280 nm; t_R_ = 6.5 min), and (−)-epicatechin (λ_max_ = 280 nm; t_R_ = 7.6 min; Figure 4). Quantification of the identified substances showed that the level of (±)-catechin (3.03 mg/(g of ADM)) was the highest in microshoots. Concentrations of epigallocatechin gallate (0.38 mg/(g of ADM)) and (−)-epicatechin (0.55 mg/[g of ADM]) were 8- and 6-fold lower, respectively, as compared to the content of (±)-catechin.

Widely used synthetic antioxidants can cause health problems [57]. Therefore, the search for natural antioxidants in the form of essential oils or plant extracts is of increasing relevance [58,59]. The antioxidant activity of *Sorbaria* plants has been reported repeatedly [60,61,62]. Pakistani scientists Izhar et al. [19] have detected high antioxidant potential in an extract from *S. tomentosa* (Lindl.); the extract Rehder can be employed to stabilize sunflower oil.

DPPH is a stable radical widely used to assess the antioxidant potential of extracts [63]. Our *S. pallasii* microshoot extract showed some DPPH radical scavenging activity (IC_50_ = 589.66 ± 13.68 µg/mL). The antioxidant activity of standard substances—trolox and ascorbic acid—proved to be higher: IC_50_ = 7.74 and 8.69 µg/mL, respectively. The antiradical activity of the microshoot extract is probably due to the relatively high content of tannins, phenolcarboxylic acids, and catechins. Catechins are generally recognized antioxidants owing to a large number of phenolic hydroxyl groups in their chemical structure. The presence of a hydroxyl group in the gallate moiety makes epigallocatechin gallate a highly effective free radical scavenger as compared to many other standard antioxidants such as ascorbic acid, tocopherol, and trolox [64,65]. In addition to the antioxidant activity, catechins possess antimicrobial, antiallergenic, anti-inflammatory, and other effects [66,67,68]. The sufficiently high concentration of catechins in the extract from *S. pallasii* microshoots indicates good prospects for its further biotechnological and phytochemical studies.

## 4. Conclusions

This study describes for the first time the successful introduction to culture in vitro of *S. pallasii*, which is an endemic species of the Far East and Siberia. Aseptic culture was established by surface sterilization of flower buds with 0.2% HgCl_2_ solution and subsequent cultivation of each explant in an individual vial. The best quality and quantity (5.7 ± 1.2 per explant) of microshoots were observed when the plant material was cultivated on MS agar-solidified medium supplemented with only a cytokinin (5.0 µM BA) as a PGR. Nonetheless, further investigation and optimization of the in vitro culture conditions are needed for the species under study. *S. pallasii* can become a promising source of biologically active compounds. Phytochemical assays of the *S. pallasii* microshoot extract showed that it contains tannins, phenolcarboxylic acids, catechins, and flavonoids, all of which most likely contribute to its antioxidant activity.

## Data Availability

Raw data are available upon request.
